# Maternal serum alpha-fetoprotein levels in fetal hydrocephalus: a retrospective population based study

**DOI:** 10.1186/1471-2393-6-23

**Published:** 2006-07-07

**Authors:** Terrence P Szajkowski, Bernard N Chodirker, Karen M MacDonald, Jane A Evans

**Affiliations:** 1 Department of Biochemistry and Medical Genetics, University of Manitoba, Winnipeg, Manitoba, Canada; 2 Department of Pediatrics and Child Health, University of Manitoba, Winnipeg, Manitoba, Canada

## Abstract

**Background:**

Although maternal serum alpha-fetoprotein (MSAFP) is a highly sensitive marker for certain congenital malformations such as open neural tube and ventral wall defects, its usefulness as a screening test for fetal hydrocephalus is uncertain. We wished to determine the distribution of maternal serum alpha-fetoprotein levels associated with fetal hydrocephalus in a population-based screening program in Manitoba, and their potential relationship to additional anomalies.

**Methods:**

Cases of fetal hydrocephalus unrelated to neural tube defects were ascertained from multiple sources and reviewed. Cross-reference with the Manitoba Maternal Serum Screening Program database determined which mothers had undergone maternal serum screening. Mean MSAFP levels in both isolated and complex hydrocephalus were calculated and compared with the general population of screened pregnancies using Independent Samples T-tests.

**Results:**

Mean MSAFP levels in 70 cases of fetal hydrocephalus were significantly higher than those of the general population of screened pregnancies (*P *= 0.029). This was due to the fact that mean MSAFP levels in those cases with other major anomalies were increased over those of the general population (*P *= 0.041); cases with hydrocephalus alone showed no significant difference (P = 0.203). Only seven cases (10%) had MSAFP levels ≥ 2.3 multiples of the median, the cut-off used in Manitoba. However, six of these (86%) had additional major and/or minor malformations.

**Conclusion:**

MSAFP screening has low sensitivity for fetal hydrocephalus and is rarely elevated in isolated cases. However, when fetal hydrocephalus is detected, elevated MSAFP levels indicate that the fetus is at significant risk to have additional malformations and further investigations, including chromosome breakage studies, may be indicated.

## Background

Although maternal serum alpha-fetoprotein (MSAFP) is a highly sensitive marker for certain congenital malformations such as open neural tube and ventral wall defects, its usefulness as a screening test for fetal hydrocephalus is uncertain. Since Seppala and Unnerus [1] first noted the possible association between elevated amniotic fluid alpha-fetoprotein (AFAFP) levels and fetal hydrocephalus, there have been relatively few reports of AFP levels in such cases. The purpose of this retrospective, population-based study was to determine the relationship between MSAFP levels and fetal hydrocephalus in women undergoing routine maternal serum screening.

## Methods

After approval had been obtained from the University of Manitoba Health Research Ethics Board, multiple sources including the Manitoba Maternal Serum Screening Program (MMSSP) pregnancy outcomes database, the Section of Genetics and Metabolism (now the Genetics and Metabolism Program of the Winnipeg Regional Health Authority) records and the Health Sciences Centre, Winnipeg patient database were utilized to ascertain all cases where hydrocephalus had been diagnosed prenatally or shortly after birth (termed fetal hydrocephalus) and where the mother's estimated date of delivery had been between January 1, 1985 and December 31, 2001. The records of the MMSSP were then used to determine in which of these cases maternal serum screening had been performed on the mother. The year 1985 was chosen as the start date for ascertainment because that was the year in which the MMSSP (formerly the Manitoba Maternal AFP Screening program) became a formal provincial program, providing screening on request to all residents at no charge. Chart review of cases also documented information on additional malformations so that MSAFP analyses on the subgroups of both isolated and complex fetal hydrocephalus could be performed. Cases of hydrocephalus in association with a neural tube defect and those in multiple gestation pregnancies were excluded from the study because of the known associations with elevated MASFP.

The control group used in the MSAFP analysis included all singleton pregnancies screened by the MMSS program in Manitoba between 1990 and 2001 inclusively (1990 was the first year that computerized records were kept), in which the mother was not known to be diabetic. Mothers with insulin-dependent diabetes mellitus were removed from the control group to allow easier comparison with other studies, many of which use different methods to control for the slower rise in MSAFP in women with this condition.

The SPSS 11.5 Independent-Samples T-test (also known as the two sample t-test) was used to compare the mean MSAFP values of all cases and controls. Mean MSAFP levels for both the subgroup of cases of complex fetal hydrocephalus and the subgroup of cases of isolated fetal hydrocephalus were also compared to the control group. In addition, mean MSAFP levels in complex and isolated cases were compared. MSAFP levels are reported in multiples of the median (MoM) so values can theoretically range from zero upward, with the expected median being about 1.0 MoM. Because of this, distributions derived from MSAFP data are characteristically skewed to the right. To normalize the distributions and allow use of the parametric T-test, the log of the MoM values of all MSAFP data was used in the analysis. The sensitivity of MSAFP screening as a predictor for fetal hydrocephalus was established by calculating what proportion of screened cases fell at or above 2.3 MoM.

## Results

Between 1998 and 2001 inclusively, there were 281,288 livebirths and stillbirths in Manitoba, while 155,518 pregnancies or approximately 55% were screened by MSAFP. A total of 131 cases of fetal hydrocephalus with no associated neural tube defect were ascertained, giving a minimal incidence of 4.66/10,000 total births. Of the 131 cases, 73 (56%) had MSAFP data available. Three cases with MSAFP data were excluded from the study due to twin pregnancies leaving 70 for analysis. Of these, 40 cases were of complex fetal hydrocephalus with additional major and/or minor malformations identified, while 30 cases were of isolated hydrocephalus.

Four different T-tests were performed (Table 1). The first, comparing the MSAFP values of all cases of fetal hydrocephalus to those of the control population, indicated a significant increase in the hydrocephalus cases. The second, comparing MSAFP levels in the subgroup of cases with complex fetal hydrocephalus to controls was also significant. The third T-test, comparing the subgroup of cases of isolated hydrocephalus to the control population, and the fourth, comparing complex and isolated fetal hydrocephalus cases, showed no significant differences.

**Table 1:** Group statistics and T-test results of MSAFP analysis

**T-test**	**N**	**Mean MoM**	**Mean of Log values**	**T-test P-value**
All cases Controls	70 91,541	1.34 1.14	0.0727 0.0231	**0.029**
Complex cases Controls	40 91,541	1.44 1.14	0.0910 0.0231	**0.041**
Isolated cases Controls	30 91,541	1.20 1.14	0.0484 0.0231	**0.203**
Isolated cases Complex cases	30 40	1.20 1.44	0.0484 0.0910	**0.198**

In Manitoba, MSAFP values of 2.3 MoM or greater are currently considered elevated and warrant fetal assessment. Of the cases of fetal hydrocephalus that had MSAFP testing performed, 10.0% (7 cases) fell at or above this cut-off. These seven cases are summarized in Table 2.

**Table 2:** Summary of cases with elevated MSAFP levels

**Case number**	**MSAFP levels (MoM)**	**Associated findings**
1	4.3	Patent ductus arteriosus, patent foramen ovale, dysmorphic skull, low set left ear
2*	2.3	Cystic hygroma, left pleural effusion, cardiac anomalies (unspecified), abnormal limb position, oligohydramnios
3	3.2	Epicanthic folds, hypertelorism, short nose with a long philtrum, hypoplastic toenails
4**	2.9	Cerebral dysgenesis, ascites, cardiac hypertrophy, amputation of both hands and feet, ambiguous external genitalia
5***	2.7	Esophageal atresia, tracheo-esophageal fistula, hemivertebrae, absent septum pellucidum, choroid plexus cyst
6***	2.6	Agenesis of corpus callosum, hypoplastic cerebellum, abnormal cerebrum, down slanting palpebral fissures, microphthalmia, micrognathia, thin distally placed thumbs, esophageal atresia, tracheo-esophageal fistula, C7 hemivertebrae, thoracic scoliosis, abnormal lung lobation, large left liver lobe, bilateral renal dysplasia with hydronephrosis and hydroureters, sacral dimple
7	2.5	None

* Final diagnosis: trisomy 21

** Final diagnosis: amnion disruption sequence

*** Final diagnosis: VACTERL-H syndrome

## Discussion

Since Seppala and Unnerus [1] first suggested a possible association between elevated AFP levels and fetal hydrocephalus, there has been a relative lack of information about the subject in the literature. Most of the information is in case reports based on few patients [2-14]. One of the strengths of the present study is that its focus is a large, population-based sample of cases of fetal hydrocephalus.

Seppala and Unnerus [1] postulated that the increase in amniotic fluid alpha-fetoprotein levels that they observed in four cases of fetal hydrocephalus might be due to passage of AFP-containing cerebral spinal fluid across the greatly thinned fetal skull into the amniotic cavity. In such cases, excess AFP in the amniotic fluid might pass into the maternal serum by diffusion across the placenta and fetal membranes. However, none of their patients had elevated MSAFP levels and at least one other report [3] failed to document even an elevation of AFAFP.

An alternative hypothesis would be that elevations of MSAFP in fetal hydrocephalus may be due to other undetected malformations. Previously, we reported four infants in whom hydrocephalus was identified during a fetal assessment performed for elevated MSAFP and who were found at birth to have VACTERL-H or other patterns of multiple anomalies [14]. (VACTERL-H is a heterogenous condition where hydrocephalus occurs in conjunction with two or more anomalies seen in the Vertebral-Anal-Cardiac-Tracheo-Esophageal-Renal-Limb association. Although many cases are apparently sporadic, both sex-linked and autosomal recessive forms exist and excessive chromosome breakage has been documented in both.) Esophageal atresia was present in all four of these cases, but not identified until birth in any of them. This malformation was singled out as a possible cause of the MSAFP elevations as we had previously documented an association between esophageal atresia and elevated MSAFP and hypothesized that impaired swallowing might prevent reabsorption of AFAFP[15]. Impaired renal function is another potential cause of elevated MSAFP and several sets of siblings with both congenital nephropathy and hydrocephalus or ventriculomegaly have been reported [16]; [17]; [18]. In cases of this apparently autosomal recessive condition, MSAFP levels have consistently been extremely high (>8.0 Mom).

Our present study has determined that, on a population basis, MSAFP levels are significantly higher in cases of fetal hydrocephalus than in the general population of screened pregnancies. Repeating this analysis using subgroups of isolated and complex hydrocephalus reveals that only complex cases show a significant increase in mean MSAFP levels, suggesting that the elevation in MSAFP levels may well be due to the other malformations present in some cases.

These data represent the first population-based analysis of MSAFP levels in fetal hydrocephalus and confirm that, as suggested by the previous case reports, mean MSAFP levels are significant higher in this group of malformed fetuses. The more pertinent statistic from a clinical standpoint is sensitivity. In the present study, only 10% of cases had had values ≥ 2.3 MoM, our current cut off for further investigation, which is even lower than the 25% sensitivity reported by Burton [19]. However, six of our seven cases had associated major and/or minor malformations and two had obvious VACTERL-H with esophageal atresia among their spectrum of anomalies.

The most likely explanation for high MSAFP in a pregnancy where fetal hydrocephalus is detected is spina bifida. However, if this is ruled out, these fetuses remain at risk to have additional malformations and should be monitored closely should the pregnancy be continued. Fetal hydrocephalus is normally considered a high risk situation. Our results indicate that the concomitant finding of elevated MSAFP warrants consideration of chromosome breakage studies in addition to standard karyotyping as Fanconi anemia [20]; [21], X-linked VACTERL-H [22] and Nijmegen breakage syndrome [23] may all present with both excess breaks and hydrocephalus. As well, detailed serial fetal assessments and detailed neonatal evaluation should be performed as some malformations such as esophageal atresia can be difficult to detect while others such as hydronephrosis or mild radial ray deficiency may only be evident later in gestation or postnatally. Awareness that a syndromal diagnosis is possible may help ensure that the family receives appropriate counselling with respect to prognosis and recurrence risk.

## Conclusion

MSAFP screening has low sensitivity for fetal hydrocephalus and is rarely elevated in isolated cases. However, when fetal hydrocephalus is detected, elevated MSAFP levels indicate that the fetus is at significant risk to have additional malformations and further investigations, including chromosome breakage studies, may be indicated.

## Competing interests

The author(s) declare thay they have no competing interests.

## Authors' contributions

TS collected and carried out the preliminary analyses of the data as part of his thesis work for an MSc at the University of Manitoba. Currently a medical student at the University of Manitoba, he wrote the first draft. BC is clinical director of the Manitoba MSS program and was responsible for manipulating its databases including extracting the appropriate control population for statistical analysis, and for critically reviewing the manuscript. KM is coordinator of the MMSSP. Her earlier work on elevated MSAFP and VACTERL-H was critical with respect to the design of this project. She was also responsible for reviewing pregnancy outcomes for all elevated cases. JAE was TS's thesis supervisor and was primarily responsible for the design and implementation of the research project as well as for securing the necessary funding. She wrote and submitted the final draft.

## Pre-publication history

The pre-publication history for this paper can be accessed here:



## References

[B1] Seppala M, Unnerus HA (1974). Elevated amniotic fluid alpha fetoprotein in fetal hydrocephaly. Am J Obstet Gynecol.

[B2] Seppala M (1975). Fetal pathophysiology of human alpha-fetoprotein. Ann N Y Acad Sci.

[B3] Williamson EM, Siggers DC, Miller JF (1976). Amniotic fluid alpha-fetoprotein and congenital hydrocephalus. Br Med J.

[B4] Gordon YB, Grudzinskas JG, Kitau MJ, Usherwood MM, Letchworth AT, Chard T (1978). Fetal wastage as a result of an alpha-fetoprotein screening programme. Lancet.

[B5] Henrion R, Herbinet E, Cedard L, Dallot E, Sender A, Rouvillois JL (1978). [Hydramnios and fetal malformations. Use of estimating the level of alpha-fetoprotein and of bilirubin in the amniotic fluid during the last trimester of pregnancy (author's transl)]. J Gynecol Obstet Biol Reprod (Paris).

[B6] Klink F, Grosspietzsch R, Conte N, Vallerino G, Diani F (1981). Alpha-fetoprotein and beta-human chorionic gonadotropin in amniotic fluid. Int J Biol Res Pregnancy.

[B7] Robertson RD, Sarti DA, Brown WJ, Crandall BF (1981). Congenital hydrocephalus in two pregnancies following the birth of a child with a neural tube defect: aetiology and management. J Med Genet.

[B8] Laurence KM, Dew JO, Dyer C, Downey KH (1983). Amniocentesis carried out for neural tube indications in South Wales 1973–1981: outcome of pregnancies and findings in the malformed abortuses. Prenat Diagn.

[B9] Augier D, Sarramon MF, Rorive C, Dutau G, Sablayrolles B, Reme JM (1985). [Results of an experiment in systematic prenatal screening for neural tube malformations by assay of alpha fetoproteins in dried blood samples (12,480 pregnancies)]. J Genet Hum.

[B10] Thomas RL, Blakemore KJ (1990). Evaluation of elevations in maternal serum alpha-fetoprotein: a review. Obstet Gynecol Surv.

[B11] Sarandakou A, Kassanos D, Phocas I, Kontoravdis A, Chryssicopoulos A, Zourlas PA (1992). Amniotic fluid hormone profiles during normal and abnormal pregnancy. Clin Exp Obstet Gynecol.

[B12] Goodburn SF, Yates JR, Raggatt PR, Carr C, Ferguson-Smith ME, Kershaw AJ (1994). Second-trimester maternal serum screening using alpha-fetoprotein, human chorionic gonadotrophin, and unconjugated oestriol: experience of a regional programme. Prenat Diagn.

[B13] Walker ME, Lynch-Salamon DA, Milatovich A, Saal HM (1996). Prenatal diagnosis of ring chromosome 6 in a fetus with hydrocephalus. Prenat Diagn.

[B14] MacDonald KM, Chodirker BN, Evans JA (1999). Elevated maternal serum alphafetoprotein in VACTERL-H. Am J Hum Genet.

[B15] Chodirker BN, Chudley AE, MacDonald KM, Harman CR, Evans JA (1994). MSAFP levels and oesophageal atresia. Prenat Diagn.

[B16] Sinclair-Smith CC, Wiggelinkhuizen J, Nelson MM (1980). Increased amniotic fluid alpha-fetoprotein, familial hydrocephalus, and renal dysmorphology. Am J Dis Child.

[B17] Reuss A, den Hollander JC, Niermeijer MF, Wladimiroff JW, van Diggelen OP, Lindhout D (1989). Prenatal diagnosis of cystic kidney disease with ventriculomegaly: a report of six cases in two related sibships. Am J Med Genet.

[B18] Jolly M, Goodburn S, Cox P, Loughna P (2003). Congenital nephropathy and ventriculomegaly: a report of four cases. Prenat Diagn.

[B19] Burton BK (1988). Outcome of pregnancy in patients with unexplained elevated or low levels of maternal serum alpha-fetoprotein. Obstet Gynecol.

[B20] Rossbach HC, Sutcliffe MJ, Haag MM, Grana NH, Rossi AR, Barbosa JL (1996). Fanconi anemia in brothers initially diagnosed with VACTERL association with hydrocephalus, and subsequently with Baller-Gerold syndrome. Am J Med Genet.

[B21] Cox PM, Gibson RA, Morgan N, Brueton LA (1997). VACTERL with hydrocephalus in twins due to Fanconi anemia (FA): mutation in the FAC gene. Am J Med Genet.

[B22] Wang H, Hunter AG, Clifford B, McLaughlin M, Thompson D (1993). VACTERL with hydrocephalus: spontaneous chromosome breakage and rearrangement in a family showing apparent sex-linked recessive inheritance. Am J Med Genet.

[B23] Muschke P, Gola H, Varon R, Ropke A, Zumkeller W, Wieacker P (2004). Retrospective diagnosis and subsequent prenatal diagnosis of Nijmegen breakage syndrome. Prenat Diagn.

